# Province-Wide Stool Color Card Screening for Biliary Atresia in Lower-Saxony: Experiences with Passive Distribution Strategies and Results

**DOI:** 10.3390/ijns7040075

**Published:** 2021-11-04

**Authors:** Omid Madadi-Sanjani, Joachim F. Kuebler, Marie Uecker, Eva-Doreen Pfister, Ulrich Baumann, Berit Kunze-Hullmann, Jochen Blaser, Thomas Buck, Claus Petersen

**Affiliations:** 1 Department of Pediatric Surgery, Hannover Medical School, 30625 Hannover, Germany; kuebler.joachim@mh-hannover.de (J.F.K.); uecker.marie@mh-hannover.de (M.U.); petersen.claus@mh-hannover.de (C.P.); 2 Division of Pediatric Gastroenterology and Hepatology, Department of Pediatric Kidney, Liver and Metabolic Diseases, Hannover Medical School, 30625 Hannover, Germany; pfister.eva-doreen@mh-hannover.de (E.-D.P.); baumann.u@mh-hannover.de (U.B.); 3 Liver Unit, Birmingham Women’s and Children’s Hospital, Institute of Immunology and Immunotherapy, University of Birmingham, Birmingham B4 6NH, UK; 4 Leberkrankes Kind e.V., National German Patient Organization for Diseases of the Liver in Children, 20038 Hamburg, Germany; berit.hullmann@gmail.com; 5 Representative Office of Lower Saxony, Techniker Krankenkasse (Health Insurance), 30159 Hannover, Germany; jochen.blaser@tk.de; 6 Medical Association of Lower Saxony, Representative Office of Hannover, 30625 Hannover, Germany; dthomasbuck@aol.com

**Keywords:** biliary atresia, screening, Kasai portoenterostomy

## Abstract

Background: Stool color card (SCC) screenings for biliary atresia (BA) have shown to improve Kasai timing and outcome significantly. Both obligatory and non-obligatory screenings with passive distribution strategies have proven to be effective. Therefore, we have initiated a voluntary SCC program and aim to describe our experience. Methods: Since 2017 we supply all maternity wards in Lower-Saxony with SCC. Attending pediatricians and parents of BA infants were contacted via questionnaires and asked for their evaluation of the SCC screening. Results: 85.2% of attending pediatricians support the SCC screening, but only 78.1% considered the initiative useful. In their clinical routine, only 67% of visiting parents report to have received an SCC at the maternity hospital. In the group of parents of BA infants, only 54% (7/13) had received an SCC. Out of those seven parents, only one had referred their child to a children’s hospital based on pathological SCC results. The lack of SCC education in the maternity hospitals was made responsible by parents. Within three years, only one infant with BA was identified through the SCC. Conclusions: Our voluntary SCC screening shows serious limitations with inacceptable distribution of SCCs and low acceptance of attending pediatricians. SCC programs in decentralized health care systems without educational campaigns, standardized diagnostic and treatment algorithms and the definition of reference centers are additional burdens for local health care providers without the promised benefit.

## 1. Introduction

For infants with biliary atresia (BA) the Kasai procedure (KPE) offers a limited chance for survival with native liver. While only a few prognostic factors for the KPE outcome are known, numerous studies have confirmed that an early diagnosis and treatment is significantly correlated with an improved native liver survival [1,2].

In Asia, with the highest global BA incidence, the idea of a mass screening for biliary atresia was already discussed in the 1980s and led to the stool color card (SCC) screening model that was established in the Japanese Tochigi Prefecture in 1994 [3,4]. While pale stool is an early symptom of those patients, the screening method is simple: SCC include photographs of multiple stool colors, highlighting pathological results [3]. In Japan the SCC are distributed antenatal to parents with the Maternal and Child Health Handbook by the local government [3]. Since implementation of the screening, the age at diagnosis and Kasai procedure in this region decreased significantly, resulting in an improved survival with native liver and reduced transplant numbers [3,5]. Since then, numerous SCC screenings were implemented with differing protocols. While governmental-led obligatory programs were implemented in Taiwan, Chinese provinces and Switzerland, voluntary programs with passive distribution strategies were established in Canada, Italy and Portugal [5,6,7,8,9,10]. However, all programs have in common, that they reported on earlier Kasai-procedures [11]. Those studies were complemented by cost- and health-effectiveness analysis, suggesting that SCC screenings in countries with high and low BA incidences not only improve BA outcomes, but additionally include savings in terms of healthcare expenditure [11,12].

Based on the international experiences with SCC screenings, we implemented a voluntary SCC program in the province of Lower Saxony. A health insurance provider financially supported production and distribution of the SCC to all maternity hospitals. In a recent analysis 98% of the maternity hospitals reported an active participation in the SCC screening [13]. While current reports mainly focus on the patients outcome with SCC programs, we identified a lack of information on SCC handling for the voluntary programs with passive distribution strategies. Furthermore, no reports on the experiences of attending pediatricians with SCC, the first-line advisors for parents and the initiators of further diagnostics, are available.

Therefore, our aim was to evaluate the perspectives of attending pediatricians on a voluntary SCC screening and to review three years of our SCC program based on the BA infants born in the province of Lower Saxony during the study period.

## 2. Patients and Methods

### 2.1. Screening Strategy in Lower Saxony

In 2016 we performed our first analysis and identified 76 maternity hospitals in Lower Saxony. All maternity wards were supplied with SCC and information on the screening methodology and its aim [13]. The screening was started in January 2017 and simultaneously we contacted and involved the regional midwifes association and we started educational programs for regional practitioners and pediatricians. We annually printed approximately 850,000 SCC and all maternity wards were instructed to contact the principal investigators, if additional SCC were necessary. In 2019 we started to additionally supply midwifery out-patient clinics with SCC.

**Figure 1:**
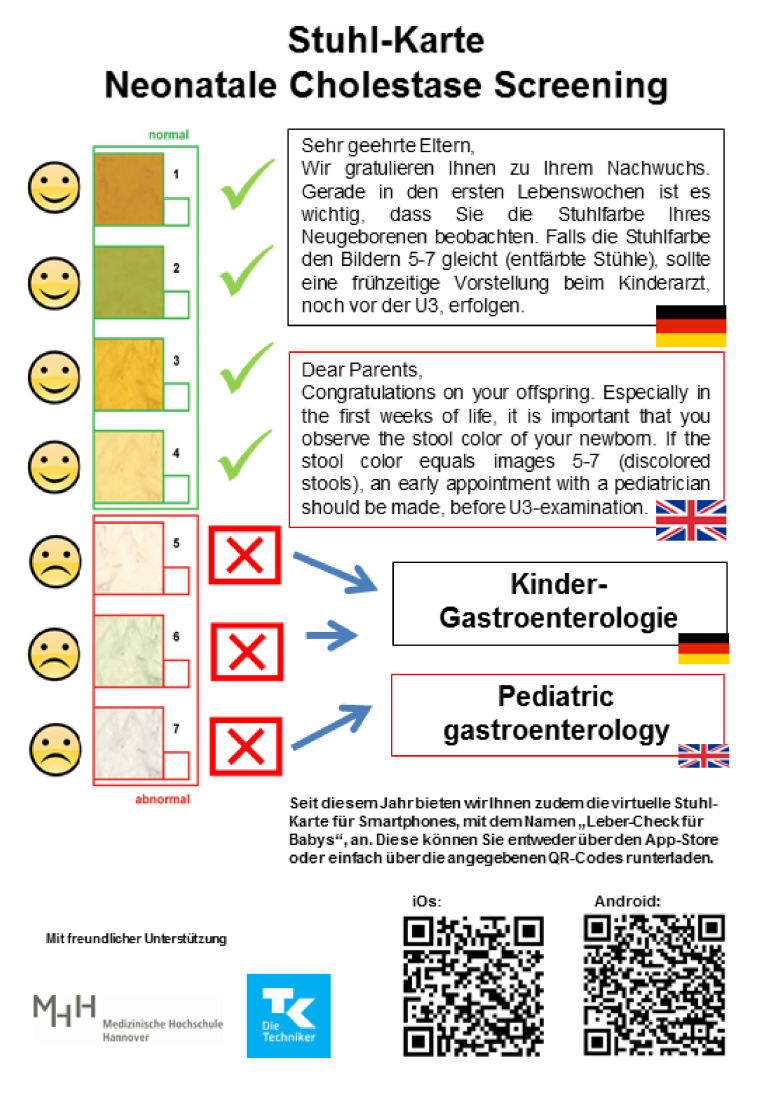
The stool color card of the Lower-Saxony pilot project.

In 2021 the SCC was revised, including now QR codes for a digital SCC for smartphones. The smartphone application “Lebercheck fuer Babys” was financially supported by the German patient’s initiative “Leberkrankes Kind e.V.” (BKH) and the health insurance company “Techniker Krankenkasse” (JB) [Figure 1].

### 2.2. Questionnaire for Attending Pediatricians in Lower Saxony

First, we aimed to evaluate the attending pediatricians perspective on the SCC screening using a semistructured questionnaire including 30 questions on their experiences of the previous three years, the regional distribution rates, the applicability of the SCC, pathological SCC results and their consequences during the observational period and diagnostic algorithms in cases of the neonatal cholestasis in their outpatient clinics.

Due to the German data protection act we were not able to get a full list of all attending (out-patient) pediatricians in Lower-Saxony. Therefore, in cooperation with the medical council of Lower Saxony, all attending pediatricians in the region of Hanover/Hildesheim and Wilhelmshaven were contacted using an email distribution list. The pediatricians were asked to answer the questionnaire, which was accessible through the SurveyMonkey^®^ online survey system. The survey consisted of multiple choice and free text questions and was open for response from April to July 2020. The estimated response time was between 8 and 20 min. Answers of the survey were anonymous and therefore could not be traced. Four weeks after the first call, all participants received a reminder e-mail. The percentages given for evaluation were calculated on the basis of the number of responses received. We did not perform separate statistics, as descriptive analyses are automatically generated in SurveyMonkey^®^ (means, medians and percentages).

### 2.3. Questionnaire for Parents of Infants with Biliary Atresia from Lower Saxony

For the evaluation of the experiences of parents of BA infants, born in Lower Saxony during the study period, we performed a retrospective chart review and identified all patients with BA from Lower Saxony. All parents had given written consent during their in-patient stay at the start of treatment, to be actively contacted for the purpose of scientific studies. Parents were supplied with a questionnaire and a copy of the stool color card [Figure 1]. They were asked whether they received an SCC in the maternity wards and if they have used it. Parents who did not receive an SCC were asked, if the card is practicable and helpful, and whether they personally consider a stool color card screening useful.

### 2.4. Patients and Public Involvement

A deputy of the patients initiative “Leberkrankes Kind e.V.” (BKH) contributed to the screening pilot project, the development of the parents survey and coauthored this paper.

## 3. Results

### 3.1. Survey of Attending Pediatricians in Lower Saxony

#### 3.1.1. Experiences with the Stool Color Cards in Out-Patient Clinics

A total of 57 attending pediatricians from the selected regions of Lower Saxony responded to the survey.

From the perspective of the majority of pediatricians (85%), since the implementation of the SCC screening, it has become part of the routine care of infants. However, only two-thirds of parents reported during the first regular check-ups of their child that they had received an SCC with their Child Health booklet in the maternity hospital. Half of those parents brought the SCC to the first check-up to the outpatient clinic. Ten percent of pediatricians reported that parents had come to the out-patient clinic earlier or unscheduled, because of the SCC. Those pediatricians felt that the SCC could make parents feel insecure and possibly lead to additional, unnecessary diagnostics. Furthermore, 26% of respondents reported that through the additional parental consultations or diagnostics, they experienced increased work-load in their clinical routine.

#### 3.1.2. Diagnostic Algorithms for Abnormal Results

Sixty-eight percent of the contacted attending pediatricians reported that they would check the serum bilirubin in a jaundiced child with pale stools, while 16% would immediately refer such infants to a children’s hospital. Six percent of respondents would at least perform an additional serum bilirubin test within a short interval, before referring newborns to a pediatric gastroenterologist. Thirty percent of respondents would refer jaundiced children immediately to a specialized pediatric liver unit.

### 3.2. Results of the SCC Screening in Out-Patient Clinics

Nine percent of respondents initiated further diagnostics based on results of the SCC.

Twenty-one cholestatic infants have been referred to children’s hospitals for further diagnostics within the three years, based on the SCC results. Slightly more than half of these children (12/21) were younger than 30 days of life and one-third were younger than 45 days (7/21) when they were referred to hospitals for further work-up. At referral, only two neonates (9.5%) were over 60 and one was older than 90 days of life. Out of the 21 children, one was diagnosed with biliary atresia.

Alpha-1 antitrypsin deficiency was found in in eight children (38.1%), five had neonatal hepatitis (23.8%), four had cystic fibrosis (19.0%), and three children had Alagille syndrome (14.3%). Out of these children, 71% are now in regular follow-up in specialized liver units.

### 3.3. Evaluation of the SCC Screening by Attending Pediatricians

Only 78% of respondents considered a mass screening for neonatal cholestasis to be useful. In fact, 70% of attending pediatricians reported that neonatal cholestasis is of low relevance in their daily practice. Nine-teen percent do not support a continuation of the SCC screening.

Those attending pediatricians consider a screening too expensive and criticize the focus on such a rare disease. In their opinion, SCC are superfluous for the simple reason that both midwives and parents could identify acholic stools without additional tools.

### 3.4. Survey of Parents of BA Patients Born between 2017 and 2020 in Lower Saxony

Based on the numbers from the German Federal Statistical Office, the annual birth rate in Lower Saxony was approximately 73,520 newborns per year from 2017 to 2020 [14]. The authors in Leonhardt et al. estimated a BA incidence in Germany of 1:19.049 live births per year [15]. Therefore, we expect 3.9 BA cases in Lower Saxony per year. During the 3.5 year study period, 13 patients (3.7 BA cases/year) with BA underwent Kasai procedure in the Hannover Medical School, with no primary liver transplantation during this period. We expect to have covered all BA cases in the province of Lower-Saxony.

### 3.5. SCC Supply and Education at the Maternity Hospitals

Out of the thirteen parents, only seven (53.8%) were given an SCC in the Child Health booklet in the maternity hospital. None of those parents had received any education regarding the importance of the stool color within their child’s first weeks of life. Only one (7.7%) of the parents reported that the SCC had led to an earlier presentation of the infant at an attending pediatrician. This child underwent the Kasai procedure at 52 days of life and is jaundice-free with its native liver at the 18-months follow-up. The additional six parents (92.3%) reported that better education would have led to an earlier presentation and thus probably to an earlier diagnosis.

### 3.6. Timing of Referral to a Children’s Hospital

Out of the thirteen infants, three (23.1%) were referred to a children’s hospital before 45 days of life. Seven infants (53.8%) were between 45 and 60 days of life at referral, and another three (23.1%) were older than 60 days at admission, while one (7.7%) was already 90 days of life. The reasons for the delay of further diagnostics were the inadequate education on the infant’s stool color within the first weeks of life, and the misinterpretation of the infant’s stool color by midwifes and attending pediatricians—blaming breastmilk induced icterus for the child’s condition.

## 4. Discussion

Based on the excellent results in the literature, including obligatory and non-obligatory stool color card (SCC) screening programs with passive distribution strategies, we have started a pilot project for a SCC screening in Lower Saxony in 2017 [Table 1] [7,16]. Within the three years of our SCC project, only one infant was diagnosed for BA based on the SCC results. Therefore, we had to conclude that our voluntary program failed to achieve the same results as in the literature. We identified numerous problems in our screening project, starting with the educational activities. Data on the sensitivity of SCC in the primary screening phase, ranging from 50% to 72.5% in the literature, already shows us that a learning process needs to be included into the evaluation [7,17]. Continuous educational programs for parents, midwives and attending pediatricians are much more crucial, than the simple distribution of SCC. Results from Taiwan have shown, that this educational process can improve the SCC sensitivity to 97% [17]. Furthermore, the interviews of the parents and attending pediatricians reveals the non-standardized processes following a pathological SCC result.

Those missing predefined screening structures in our pilot project have led to reservations of attending pediatricians regarding a BA mass screening. Despite of the doubts regarding the necessity of a screening, knowing the low incidence of BA in the Western hemisphere, attending pediatricians express the concern of additional work-load in their routine. In contrast, the screening program in Taiwan includes a stool color card registry with specialized personnel that can be contacted for any case of abnormal stool color and are obligated to give recommendations for the further work-up within 24 h [17,18]. Those study centers with specialized personnel could be a massive relief for local health care providers and shifts the responsibilities to the specialized centers.

Furthermore, attending pediatricians argued that SCC could unsettle parents, leading to numerous presentations in out-patient clinics and potentially resulting in unnecessary diagnostics. However, the authors in Borgeat et al. recently presented the results of a survey of parents, showing that the vast majority considered the SCC helpful and simple, while only the minority reported on uneasiness using the SCC [20]. While the study group from Geneva faced similar reservations of Swiss physicians, blaming the SCC for parents anxiety, the authors concluded the exact opposite, emphasizing the benefits of an SCC screening [20].

Despite our report on the serious limitations of the SCC pilot project for biliary atresia in Lower Saxony, we observed a beneficial accompanying identification of other cholestatic diseases through the SCC in the neonatal period. This additional benefit of an SCC screening has been neglected in the literature, because of the crucial role of early diagnosis in biliary atresia.

So the question remains: Can we create the structures for a nationwide screening with our current experiences? Yes, but with multiple restrictions. The Federal Joint Committee, a governmental institution responsible for the evaluation of screening methods, needs to take the SCC screening in charge. The SCC should be produced centrally and should already be included in the Child Health Booklet before distribution to the maternity hospitals. At the infant’s second obligatory check-up, between the third and tenth day of life, physicians need to educate parents on the importance of stool colors, ideally using the SCC. An SCC registry needs to be established to help the local health care providers in their decision-making. Finally, the treatment structures need to be standardized. The German Association for Pediatric Surgery is currently working on a list of reference centers for the treatment of BA in Germany. Those suggested five centers or networks offer expertise in hepatobiliary pediatric surgery, pediatric hepatology, a pediatric transplant team and pathologists specialized in pediatric cholangiopathies. Furthermore, the German Society for Pediatric Gastroenterology and Nutrition (GPGE) is finalizing a guideline for the diagnostic algorithms in neonatal cholestasis including the recommendation for further diagnostics in infants with persistent jaundice after two weeks of life. These developments might result in a non-governmental centralization of BA care in Germany. 

Our report has some important limitations. We only received limited access to the contact data of regional attending pediatricians in Lower Saxony. Furthermore, while we expected to have treated all infants with BA in Lower Saxony during the observational period, it is possible that children with BA born in Lower Saxony have been treated in other German provinces. Most important, the main weaknesses is the small cohort and the response rate for both questionnaires. However, we expect to have achieved a representative overview of perspectives regarding the province-wide pilot project.

In conclusion, the results of our survey are disappointing but not discouraging. Simply implementing an SCC screening with passive distribution strategies is ineffective in a decentralized health care system like Germany. Before discussing an obligatory nationwide screening, management strategies should be standardized, including the definition for patients selection for further diagnostics, diagnostic algorithms and recommendations for reference centers. Otherwise, the internationally already validated benefits of an SCC screening will not be achieved in the current German health care structures.

## Figures and Tables

**Table 1:** Current SCC programs and their strategies of distribution and assessment. SCC, stool color card.

Country/Region	SCC Type	Distribution	Assessment
Taiwan [17]	6 photographs stool samples (3 pathological images)	Postnatally at maternity hospitals	Telephone and fax numbers for consultation were printed on the card Parents, guardians and medical personnel were instructed to inform the stool color card registry center if abnormal stool colors were noticed Professional personnel in stool card center respond to incoming referrals within 24 h Instructions and follow-ups recommentdations were given for every reported case
Japan [3]	7 photographs of stool samples (3 pathological images)	Antenatally, in the Maternal and Child Health Handbook	Parents received the SCC already prenatally Mothers were asked to fill in the corresponding number of the image on the SCC that resembled the color of her infant’s stool Results were discussed during the 1-month health check up The Department of Pediatrics at the Jichi Medical University in Tochigi Prefecture was notified of all positive cases
Canada/Quebec [16]	6 photographs of stool samples (3 pathological images)	Postnatally, at maternity hospitals	Parents were asked to monitor the infants stool color and to complete and return the SCC when their infant was 21 days old All SCC were barcoded with a unique identifier If the stool was abnormal at any time during the first month of life, parents were advised to complete the SCC immediately and mail it back to the research team and/or to contact the study team
China/Bejing [19]	7 photographs of stool samples + 7 digitally created images (3 pathological images/photographs)	Postnatally, by trained nurses in maternal facilities	Guardians were advised to monitor the infants stool within the first four months of life closely A corresponding number on the scale should be filled in at 14 days, one months and during one to four months of life The SCC office additionally checked the babies stool color on day 42 If abnormal stools were identified, parents were advised to visit the SCC office at the Department of Newborn Screening, Bejing Obstetrics and Gynecology Hospital
Switzerland [10,20]	7 photographs of stool samples (3 pathological images)	Postnatally, by the attending pediatrician or midwife	An instructive website for parents and health-care personnel was established SCC and the infants stool color are checked during the first visit with the treating physician (usually at the age of 4 weeks) SCC data are transmitted to the coordinating center in Geneva, Switzerland
Germany/Lower Saxony [13]	7 photographs of stool samples (3 pathological images)	Postnatally, at maternity hospitals	In this voluntary program all maternity hospitals and midwifes were asked to supply parents with SCC Attending pediatricians were informed about the SCC project Control of infants stool colors during the first physician visitations are obligatory—therefore, the home-based SCC screening project complements those legal obligations
